# Risk Factors for Asthma in a Helminth Endemic Area in Bahia, Brazil

**DOI:** 10.1155/2012/796820

**Published:** 2012-08-23

**Authors:** Luciana S. Cardoso, Daniela M. Costa, Maria Cecília F. Almeida, Robson P. Souza, Edgar M. Carvalho, Maria Ilma Araujo, Ricardo R. Oliveira

**Affiliations:** ^1^Serviço de Imunologia, Hospital Universitário Professor Edgard Santos, Universidade Federal da Bahia, 40110-160 Salvador, BA, Brazil; ^2^Departamento de Ciências da Vida, Universidade do Estado da Bahia, 41.150-000 Salvador, BA, Brazil; ^3^Instituto Nacional de Ciência e Tecnologia de Doenças Tropicais (INCT-DT/CNPq), 40110-160 Salvador, BA, Brazil; ^4^Escola Bahiana de Medicina e Saúde Pública, 40290-000 Salvador, BA, Brazil; ^5^Faculdade de Farmácia, Universidade Federal da Bahia, 40.170-115 Salvador, BA, Brazil

## Abstract

Protective factors associated with atopy or asthma in rural areas include socioeconomic level, overcrowding, and helminth infection. However, little epidemiological information was originated from schistosomiasis areas. This study aimed to investigate factors associated with asthma in a schistosomiasis endemic area. A questionnaire was used to obtain information on demographics, socioeconomic, and environmental features. The ISAAC questionnaire was used to identify individuals with asthma. Parasitological exam was done in all participants and skin prick test to aeroallergens in all asthmatics. Prevalence of *Schistosoma mansoni* infection was 57.4% and *Ascaris lumbricoides,* 30.8%. Asthma was found in 13.1% of the population, and 35.1% of them had a positive SPT. Active and passive smoking was positively associated with asthma, whereas *A. lumbricoides* was negatively associated. In a schistosomiasis hyperendemic region, current infection with *A. lumbricoides* is protective against asthma. However, we cannot rule out the involvement of *S. mansoni* infection in this process.

## 1. Introduction

Asthma is the most outstanding allergic disease especially in developed countries [1, 2]. However, in developing countries the frequency of asthma is much lower, particularly in rural areas, where the prevalence of parasite infection is high [3–5]. Factors associated with protection against atopy in rural populations include reduced socioeconomic level, poor living conditions, and increased overcrowding [6]. Moreover, several studies have shown a protective role of different parasites in the development of asthma or atopy [6–10].

Studies conducted in Venezuela have shown that geohelminth infections is associated with high levels of total IgE and low levels of allergen-specific IgE, as well as suppression of allergic reactivity, which is reversible by anthelmintic treatment [11, 12]. Other authors have also shown low prevalence of positive skin prick test (SPT) for aeroallergens in endemic areas for geohelminths [7, 13] or *Schistosoma spp*. [9, 14]. We have previously reported that asthmatic individuals living in regions with high frequency of *S. mansoni* infection have a mild course of asthma [15], and that treatment for schistosomiasis leads to a worsening of the clinical manifestations of asthma [3]. It is noteworthy that in these previous studies conducted with *S. mansoni *infected patients, symptoms of asthma or frequency of positive SPT was compared to individuals from outside schistosomiasis endemic areas.

The immunological mechanisms involved with protection against the development of asthma are not well known, although it has been proposed the participation of regulatory molecules [3, 14, 16–20]. Moreover, risk or protective factors associated with asthma in helminth endemic areas remain still not well established. Despite a fair amount of epidemiological information is available on risk factors for asthma in developed countries, scant attention has been given to risk factors associated with asthma in rural communities from developing countries, especially in regions endemic for schistosomiasis.

We conducted a cross-sectional study in a rural area endemic for helminths, including *S. mansoni*, to evaluate possible risk and protective factors associated with the presence of asthma. In addition to demographic, socioeconomic and environmental factors, we also evaluated the influence of current *S. mansoni*, *A lumbricoides*, *Trichuris trichiura*, and hookworm infections with the presence of asthma in individuals living in the region.

## 2. Material and Methods

### 2.1. Subjects and Endemic Area

This study was carried out at Água Preta, a rural community in the district of Gandu, south of Bahia Brazil, 300 km distant from Salvador, the capital of the state. This village is composed of about 500 inhabitants, who live in poor sanitary conditions. Using a standard questionnaire, age, sex, income, housing conditions, and exposure to pollutants were registered. Additionally, information on water contact was obtained individually or from guardians in case of children under 10 years old. The International Study of Asthma and Allergies in Childhood (ISAAC) questionnaire, which assess the personal history of bronchial asthma and rhinitis in the past 12 months, as well as family history of atopy and concomitant pathologies, was applied to each individual aging from 6 to 50 years old. In order to exclude individuals with chronic obstructive pulmonary disease (COPD), a clinical evaluation, chest X-ray and spirometry were performed in all asthmatic individuals. None of the asthmatic individuals included in this study had COPD. Subjects who presented personal history of asthma in the past 12 months according to ISAAC questionnaire underwent a skin prick test (SPT) for aeroallergens. 

Interviews were carried out initially at the health post of the region, located at the center of Água Preta. The recruitment strategy consisted in household visits to invite subjects to the health post. During the last days of the recruitment, when the number of subjects who spontaneously went to the health post were lower, new household visits were done in order to invite and perform a home-based interview for each individual who agreed in participate in this study.

The ethical Committee of the Maternity Climério de Oliveira, Federal University of Bahia, approved the present study and informed consent was obtained from individual or their legal guardians. All participants who tested positive for parasites were treated at the end of the study. Those who had complaints of allergy were properly instructed regarding indoor environmental control and drug treatment.

### 2.2. Assessment of Water Contact

The water contact questionnaire was developed based on previous observations carried out on site about habits of the population regarding the use of local rivers. Each participant was asked to provide the frequency and duration of the exposure to the main seven activities (farming, bathing, washing clothes, washing hair, washing dishes, fishing, and playing). Subjects were classified across all activities into four categories to reflect the level of exposure: no exposure, low exposure (<1 h/week), medium exposure (1–3 h/week), or high exposure (1–3 h/day) [21].

### 2.3. Fecal Examinations for Parasites

All participants received a plastic container for fecal samples at three time points 2–30 days apart. Participants were instructed to deposit stool sample and return the container immediately to the collection point, where samples were stored at 4°C. Each stool sample had a slide prepared and tested by using the Kato-Katz method to estimate the number of *S. mansoni*, *A. lumbricoides *and *T. trichiura* eggs per gram of feces (EPG) [22]. Additionally, the Hoffmann-Pons-Janer method was used to qualitatively investigate the presence of Hookworm infection [23].

### 2.4. Skin Prick Test

SPTs were performed on all individuals identified as asthmatic according to the ISAAC criteria. Glycerinated allergens tested included *Dermatophagoides pteronyssinus*, *D. farinae*, *Blomia tropicalis*, *Periplaneta Americana,* and *Blatella germanica* (IPI-ASAC). We also used histamine (1 : 1000) and saline as positive and negative controls, respectively. SPT results were obtained 20 minutes after application, and a positive skin reaction was defined as a wheal with a mean diameter greater than 3 mm.

### 2.5. Statistical Analyses

The variable age are expressed as arithmetic mean and standard deviation (SD), whereas parasite load are expressed as geometric mean and 95% of confidence interval. Helminth infections were classified into low, moderate, or high intensity of infection according to World Health Organization (WHO) criteria [24]. Parasite load ranging from 1 to 99 epg for *S. mansoni *is classified as low, 100 to 300 epg moderate, and equal or greater than 400 epg high intensity of infection. For *A. lumbricoides *low intensity of infection is defined as 1 to 4,999 epg, moderate 5,000 to 49,999 epg, and high intensity of infection is equal or greater than 50,000 epg. Light *T. trichiura *infection is considered as parasite load ranging from 1 to 999 epg, moderate 1,000 to 9,999, and high intensity of infection equal or greater than 10,000 epg. The intensity of hookworm infection was not assessed since the Kato-Katz technique is not adequate for quantifying hookworm eggs.

Statistical analyses were performed by using the STATA statistical package version 11.2 (Stata Corp., College Station, TX). A logistic regression model was used to test association between asthma and covariates in a univariate and multivariate way. The alpha level for statistical significance was established as 0.05 for all analyses and odds ratio (OR) was obtained at a 95% of confidence interval (CI). In order to reduce the chance of type I error a *P* value adjustment was performed by the Bonferroni correction in all significant associations. Multivariate analyses were adjusted for age, gender, number of stool samples evaluated, and/or smoking status. Adjustment for age and gender was performed because these could be possible confounding factors, since depending on gender or age lifestyle could be significantly different. The adjustment for number of stool samples analyzed was done because 82.8% of subjects who had three samples evaluated were asthmatics, and it is well known that the Kato-Katz sensitivity is influenced by the number of samples evaluated, reaching a sensitivity of 91.7% for the diagnosis of schistosomiasis when four samples are analyzed [25]. For investigating the influence of helminth infections on asthma, the smoking status was taken into account since this was positively associated with asthma in this region, which could be responsible for the underestimation of the real effect that helminth infections have on asthma.

## 3. Results

### 3.1. Baseline Characteristics of the Studied Subjects

A total of 427 individuals were included in this study, being 53.2% male. The mean age was 26.9 ± 19.0 years, ranging from 1 to 92 years. Approximately 95% of participants reported an average income less than the Brazilian national minimum wage (≤$250.00) per month. Regarding the presence of pollutants 94.4% of the population uses wood burning stove and 14.5% were active smokers.

The frequency of intestinal parasites was investigated in 406 (95.1%) individuals, 214 (52.7%) of whom had only one sample analyzed, whereas 160 (39.4%) and 32 (7.9%) subjects had two and three samples evaluated, respectively. The helminth most frequently identified in the region was *S. mansoni*, affecting 57.4% of the population, followed by *T. trichiura* (36.9%), hookworm (31.8%), and *A. lumbricoides *(30.8%). Intensity of infection was assessed for *S. mansoni* (89 [75–105] epg), *A. lumbricoides *(1,946 [1,278–2,963] epg), and *T. trichiura *(120 [97–149] epg).

History of exposure to contaminated water was reported by 332 (77.8%) subjects, 34.3% of whom had a low level of exposure, 18.7% medium, and 47.0% were highly exposed. The group highly exposed was composed mainly of female (67.9%), whereas the group with low level of exposure was represented mostly by men (68.4%). Despite the evident influence of gender on the intensity of exposure, age is a factor that apparently did not affect the level of exposure. The group classified as highly exposed had a mean age of 27 ± 17 years, while the groups of medium and low level of exposure had a mean age of 26 ± 18 and 27 ± 20 (*P* > 0.05), respectively. As expected, the group that reported higher level of exposure also showed higher frequency of schistosomiasis, when compared to the nonexposed group (Table 1).

The prevalence of asthma and rhinitis was evaluated by the ISAAC questionnaire in 335 individuals, representing all subjects who were 6 to 50 years old. The prevalence of self-reported wheezing in the past 12 months (here designated as “asthma”) was 13.1%. Skin prick test for aeroallergens was performed on 37 out of 44 (84.1%) asthmatic individuals. False positive result, defined as presence of skin reaction in the negative control saline, was found in only one subject (2.3%). On the other hand, false negative result, defined as no skin reaction detected, even to histamine, was found in four (9.1%) asthmatic individuals. Among those 32 individuals who had a validated skin prick test result, 13 (35.1%) was positive for at least one allergen, whereas 19 (64.9%) of them had a negative SPT result. 

### 3.2. Risk Factors Associated with Asthma

Univariate and multivariate analysis for the association between asthma and possible risk or protector factors are presented in Table 2. Evaluated variables included gender, age, income, passive or active smoking, family history of allergy, housing quality, number of people per household, use of wood burning stoves, and level of exposure to fresh water. Multivariate analyses were adjusted for age and gender.

The frequency of asthmatic individuals living in the endemic area who are active smokers was higher (26.2%) than the prevalence of active smokers among those nonasthmatic subjects (11.8%). In order to obtain the power of this difference, we performed a sample size calculation based on these data and found that 42 individuals in the “Asthmatic” group and 272 in the “nonasthmatic” group have 76.1% power to detect a difference with a significance alpha level of 0.05.

Only active or passive smoking was positively associated with the presence of personal history of asthma in the past 12 months, even after adjustment for confounding factors (Table 2). After controlling for age and gender the prevalence of asthma was 5.45 (CI 95%: 2.09–14.24; *P* = 0.001) and 3.67 (CI 95%: 1.83–7.34; *P* < 0.001) times higher among active and passive smokers, respectively, than nonsmokers.

### 3.3. Association between Asthma and Helminth Infections

The association between helminth infection or intensity of infection and asthma is shown in Table 3. Univariate analysis indicates that hookworm infection (OR = 2.54 [1.32–4.91]; *P* = 0.005), moderate intensity of *A. lumbricoides *infection (OR = 4.01 [1.74–9.28]; *P* = 0.001), *T. trichiura *(OR = 2.18 [1.13–4.20]; *P* = 0.020), and *S. mansoni* (OR = 2.89 [1.29–6.48]; *P* = 0.010) infection were positively associated with asthma. However when adjusting for the confounding factors age, gender, number of stool samples evaluated, and smoking status, no significant association was observed between hookworm (OR = 1.41 [0.54–3.72]; *P* = 0.486), *T. trichiura* (OR = 0.95 [0.36–2.54]; *P* = 0.918), *S. mansoni* (OR = 1.44 [0.48–4.29]; *P* = 0.517), or any helminth infection (OR = 0.94 [0.19–4.56]; *P* = 0.935) and asthma. Moreover, no significant association was found between number of helminth infections and asthma (data not shown).

Multivariate analysis showed that *A. lumbricoides *infection is negatively associated with asthma (OR = 0.26 [0.07–0.94]; *P* = 0.041). When the Bonferroni correction was performed this association became nonsignificant (*P* = 0.984). Nevertheless, we found a significant association between *A. lumbricoides* infection and asthma when controlling for other helminth infections (data not shown). The association between asthma and parasite load was also assessed and we found that low (OR = 0.27 [0.07–0.94] *P* = 0.071) or moderate (OR = 0.29 [0.05–1.83]; *P* = 0.187) intensity of *A. lumbricoides *infection was not associated with asthma. Since only three individuals, all nonasthmatic, showed high intensity of *A. lumbricoides *infection in the region, we were not able to test the association between high ascariasis parasite load and asthma.

## 4. Discussion

This study assessed possible risk or protective factors associated with presence of asthma in a population living in a rural poor area of Bahia, Brazil. Our results showed that the main risk factor associated with presence of asthma is smoking. We found no association between socioeconomic variables and the development of asthma. Additionally, we demonstrated that current *A. lumbricoides* infection is associated with protection against asthma when adjusting for confounding factors.

The cross-sectional design, the use of questionnaires to access information on the presence of asthma, and risk factors were the major limitations of this study. The study design did not allow us to make causal inferences, and confounding factors may not be equally distributed between groups. However, we performed statistical analysis taking into account the confounding factors, and reverse causality is not possible for most of the risk factors evaluated. We are also aware that the use of questionnaires makes the study open to information bias since it depends on the interviewed response. Therefore, misclassification of exposure may have occurred in the present study. Also, we call attention that we did not evaluate past helminth infections and that the parasitological technique used here is not sensitive for very low parasite burden.

The differentiation between asthma and COPD was one of our major concerns when planning this study. One way to reduce this possible misdiagnose was the exclusion of individuals over 50 years of age. Many authors have reported that COPD rarely presents clinically before the 5th decade of life and that the prevalence of COPD occurs primarily in smokers over 40 years of age [26, 27]. According to the PLATINO study the prevalence of COPD in individuals aging from 40 to 49 years in a metropolitan area of Brazil is 8.4% [28]. In our study only 4 (9%) asthmatic individuals aged between 41 and 50 years old. Therefore, 91% of subjects included in this study were under 41 years old. Moreover, only 1 out of 4 (25%) asthmatic individual over 40 years of age was active smoker.

Although we did not observe association between socioeconomic variables and asthma, other authors have demonstrated that reduced socioeconomic level and overcrowding are associated with protection against atopy in a rural region [6]. This phenomenon was probably not detected in our study because the overall population was homogeneous in regard to socioeconomic level, being identified only subjects with an average income less than $500 per month. Additionally, several other studies have showed an association between smoking and risk of asthma, which is consistent with our data [29–32]. Even though this association is well known, its interpretation must be made cautiously, especially when using a questionnaire-based diagnosis of asthma. Because smokers have been reported to have more severe asthma than nonsmokers, it is expected that the use of self-reported questionnaires, such as the ISAAC questionnaire, may overestimate the risk of asthma among smokers [33]. However, this bias probably did not interfere in our study since it has been shown that individuals from endemic areas for schistosomiasis have mild asthma [15]. In order to evaluate the effect of helminth infections in the presence of asthma statistical analyses were adjusted for smoking status, given that this is an independent determinant factor for asthma. 

In the last years researchers showed an increased interest in evaluating the role of helminth infections in the development of allergic diseases. Although several studies show that in endemic areas for helminths, infection with *A. lumbricoides *or other helminths has a negative association with atopy, possibly acting as a protective factor against the development of asthma, none of these studies was conducted in endemic area for schistosomiasis [6–8, 12, 34]. Studies carried out with individuals from endemic areas infected with *S. mansoni* have showed low reactivity to the skin prick test (SPT) to aeroallergens and fewer symptoms of asthma compared to asthmatics without infection [10, 15], and an inverse correlation between SPT and presence of infection [9]. It is worth noting that none of these previous studies evaluated the association between SPT or symptoms of asthma and current *S. mansoni *infection within asthmatic subjects living in the same region. The prevalence of positive SPT to aeroallergens in asthmatic individuals included in this study was 35.1%, which is far below what is found in urban regions from Brazil [10, 35, 36]. Nevertheless, we found no significant difference in the frequency of positive SPT among asthmatics currently infected or not by any helminth (data not shown). Despite the frequency of positive skin test has been low, asthmatic subjects from these regions probably have atopic asthma, demonstrated by the presence of aeroallergen-specific IgE [10]. 

The presence of asthma was not associated with current infection by *S. mansoni* in the studied population. However, the prevalence of asthma in the site was only 13.1%, which is much lower than the frequency observed in adolescents of 13 and 14 years old from urban areas of Salvador (24.6%), Feira de Santana (21.5%) or Vitoria da Conquista (30.5%), Bahia, when using the same criteria for defining asthma [5]. The prevalence of asthma was representative of the entire study population, including individuals from 6 to 50 years old. However, when analyzing only the age of 13 and 14 years of age, the prevalence was 15%, which is very close to the frequency observed in the general population in the endemic area and still far from that observed in other centers evaluated in Bahia, Brazil [5]. This is in agreement with other study conducted in different regions of Brazil, which showed lower prevalence of asthma and rhinitis in rural than in urban areas [4].

Thus, the presence of a protective factor for the development of asthma in an endemic community for helminths including schistosomiasis is evident, which could not be explained only by the presence of current *S. mansoni* infection. The fact that these individuals inhabit a region of high endemicity, constantly exposed to *S. mansoni* reinfection, might be responsible for the low prevalence of asthma observed on site, with no significant difference in the frequency of asthma among currently infected or not. On the other hand, we found in this schistosomiasis endemic area that individuals currently infected with *A. lumbricoides *have a lower prevalence of asthma when compared to noninfected subjects, which was not observed for the presence of *T. trichiura*, hookworm, any helminth infection, or even for different intensity of infection. Although this association has been lost when performing the Bonferroni correction, it is well established that reducing type I error by *P* value adjustments increases the probability of type II error for those association that are not null. The formal premise for such adjustments is the much wider hypothesis that there is no association between any variables under observation, and that only random processes govern the variability of all observations. This “universal” null hypothesis presumes that all observed associations reflect only random variation that could be obtained particularly in empiric situations [37, 38]. Once this study evaluated associations that are not empiric, but instead evidence-based, such as the association between *A. lumbricoides* infection and asthma, we are confident that this association was not due to random variation, and therefore it would be better to take the risk of making type I error and call attention to a possible beneficial association for asthma.

Studies conducted in urban areas in Brazil have showed controversial results for the association between *A. lumbricoides *infection and atopy [8, 39]. Although no association between SPT response and *A. lumbricoides *infection was found in asthmatic individuals [39], a recent study showed that the presence of *A. lumbricoides *infection is able to reduce the prevalence of positive SPT response but do not affect the prevalence of asthma [8]. Overall, helminth infection is associated with reduced risk of asthma or atopy in regions with high frequency of parasite infections [7, 11, 13], whereas in areas with low endemicity, *A. lumbricoides *infection is associated with increased risk of atopy and asthma [40, 41]. Although *A. lumbricoides *is a recognized cause of tropical pulmonary eosinophilia, chronic infection induces a regulatory immune response which includes interleukin-10- and transforming growth factor-*β*-secreting cells [42, 43]. In addition to the suppression of allergic inflammation, activation of the regulatory immune response induced by helminths is responsible for preventing the elimination of parasites and protects the host from damage that could be caused by excessive inflammatory response [44]. Our findings support the hypothesis that exposure to *A. lumbricoides* induces a generalized suppression on the immune response, which seems to be able to reduce the risk of asthma without additive effect by helminth coinfection. This effect was not observed for other helminth infections probably because *A. lumbricoides* was the less frequently helminth found in the region, which results in less exposure and lower rates of reinfection, allowing thereby the detection of differences in asthma prevalence between individuals currently infected or not.

In conclusion, our findings indicate that smoking is the major risk factors associated with asthma in a rural population endemic for schistosomiasis. However, in rural endemic areas for helminth infections the prevalence of asthma is much lower than what is observed in urban regions, and *A. lumbricoides *infection is negatively associated with the presence of asthma within the population. Although we were unable to show a protective association between current *S. mansoni* infection and asthma, we cannot rule out the possible participation of *S. mansoni *in modulating the immune response of allergic diseases, probably because this is a hyperendemic region where individuals are constantly reinfected. This study contribute to a better understanding of the role of helminth infections in allergies. 

## Figures and Tables

**Table 1:** Frequency of *S. mansoni *infection according to the level of exposure.

	*S. mansoni*		Univariate analysis		Multivariate analysis	
	Positive	Negative	OR (95% CI)	*P*	OR (95% CI)	*P*
Level of exposure						
No contact	26 (29.5%)	62 (70.5%)	1.00		1.00	
Low	62 (55.9%)	49 (44.1%)	2.18 (1.13−4.22)	0.021	2.56 (1.22−5.34)	0.013
Medium	38 (67.9%)	18 (32.1%)	3.17 (1.41−7.15)	0.005	2.67 (1.11−6.42)	0.028
High	107 (70.9%)	44 (29.1%)	4.00 (2.11−7.61)	<0.001	3.64 (1.81−7.29)	<0.001

Note: Multivariate analysis adjusted by age, gender, and number of stool samples analyzed.

**Table 2:** Univariate and multivariate analysis for the association between asthma and possible risk or protector factors.

	Nonasthmatic	Asthmatic	Univariate analysis		Multivariate analysis	
	(*n* = 291)	(*n* = 44)	OR (95% CI)	*P*	OR (95% CI)	*P*
Gender						
Female	141 (85.4%)	24 (14.6%)	1.00			
Male	150 (88.2%)	20 (11.8%)	0.78 (0.41–1.48)	0.452		
Age (years)						
6–10	44 (83.0%)	9 (17.0%)	1.00			
11–15	48 (81.4%)	11 (18.6%)	1.12 (0.42–2.96)	0.819		
16–20	41 (91.1%)	4 (8.9%)	0.48 (0.14–1.67)	0.247		
21–30	82 (88.2%)	11 (11.8%)	0.66 (0.25–1.70)	0.386		
31–40	43 (89.6%)	5 (10.4%)	0.57 (0.18–1.83)	0.345		
41–50	33 (89.2%)	4 (10.8%)	0.59 (0.17–2.09)	0.416		
Income ($)						
<250	271 (86.9%)	41 (13.1%)	1.00		1.00	
250–500	19 (86.4%)	3 (13.6%)	1.04 (0.30–3.68)	0.947	1.14 (0.31–4.17)	0.847
>500	0	0	—	—	—	—
Active smoker						
No	240 (88.6%)	31 (11.4%)	1.00		1.00	
Yes	32 (74.4%)	11 (25.6%)	2.66 (1.22–5.81)	0.014	5.45 (2.09–14.24)	0.001
Passive smoker						
No	190 (92.7%)	15 (7.3%)	1.00		1.00	
Yes	97 (78.2%)	27 (21.2%)	3.53 (1.79–6.94)	<0.001	3.67 (1.83–7.34)	<0.001
Family history of allergy						
No	180 (87.0%)	27 (13.0%)	1.00		1.00	
Yes	65 (84.4%)	12 (15.6%)	1.23 (0.59–2.57)	0.581	1.29 (0.60–2.77)	0.508
Housing quality						
Brick house	103 (92.0%)	9 (8.0%)	1.00		1.00	
Adobe house	107 (84.3%)	20 (15.7%)	2.14 (0.93–4.92)	0.073	2.03 (0.88–4.72)	0.099
Mud house	48 (87.3%)	7 (12.7%)	1.67 (0.59–4.77)	0.337	1.77 (0.61–5.08)	0.290
Number of inhabitants						
1–3	95 (87.2%)	14 (12.8%)	1.00		1.00	
4–6	148 (88.1%)	20 (11.9%)	0.92 (0.44–1.90)	0.816	0.76 (0.35–1.64)	0.485
>6	43 (81.1%)	10 (18.9%)	1.58 (0.65–3.83)	0.314	1.40 (0.56–3.52)	0.475
Wood burning stove						
No	19 (95.0%)	1 (5.0%)	1.00		1.00	
Yes	271 (86.3%)	43 (13.7%)	3.01 (0.39–23.1)	0.288	2.83 (0.37–21.88)	0.319
Exposure to fresh water						
No contact	57 (87.7%)	8 (12.3%)	1.00		1.00	
Low	86 (93.5%)	6 (6.5%)	0.50 (0.16–1.51)	0.217	0.48 (0.15–1.50)	0.207
Medium	41 (85.4%)	7 (14.6%)	1.22 (0.41–3.62)	0.725	1.26 (0.42–3.81)	0.684
High	107 (82.3%)	23 (17.7%)	1.53 (0.64–3.64)	0.335	1.60 (0.66–3.87)	0.293

Note: Multivariate analysis adjusted by age and gender.

**Table 3:** Univariate and multivariate analysis of the association between asthma and helminth infections.

	Nonasthmatic (*n* = 291)	Asthmatic (*n* = 42)	Univariate analysis		Multivariate analysis	
	33%	45%	OR (95% CI)	*P*	OR (95% CI)	*P*
Hookworm						
Negative	189(90.9%)	19 (9.1%)	1.00		1.00	
Positive	90 (79.6%)	23 (20.4%)	2.54 (1.32–4.91)	0.005	1.41 (0.54–3.72)	0.486
*A. lumbricoides*						
Negative	187 (89.0%)	23 (11.0%)	1.00		1.00	
Positive	92 (82.9%)	19 (17.1%)	1.68 (0.87–3.24)	0.122	0.26 (0.07–0.94)	0.041
Intensity (epg)						
0	193 (89.4%)	23 (10.6%)	1.00		1.00	
1–4999	60 (88.2%)	8 (11.8%)	1.12 (0.48–2.63)	0.797	0.27 (0.07–1.12)	0.071
5000–49999	23 (67.6%)	11 (32.4%)	4.01 (1.74–9.28)	0.001	0.29 (0.05–1.83)	0.187
≥50000	3 (100%)	0	—	—	—	—
*T. trichiura*						
Negative	173 (90.6%)	18 (9.4%)	1.00		1.00	
Positive	106 (81.5%)	24 (18.5%)	2.18 (1.13–4.20)	0.020	0.95 (0.36–2.54)	0.918
Intensity (epg)						
0	175 (90.7%)	18 (9.3%)	1.00		1.00	
1–999	96 (80.7%)	23 (19.3%)	2.33 (1.20–4.53)	0.013	1.02 (0.38–2.74)	0.968
1000–9999	7 (87.5%)	1 (12.5%)	1.39 (1.61–11.9)	0.765	0.30 (0.01–8.03)	0.474
≥10000	1 (100%)	0	—	—	—	—
*S. mansoni*						
Negative	113 (93.4%)	8 (6.6%)	1.00		1.00	
Positive	166 (83.0%)	34 (17.0%)	2.89 (1.29–6.48)	0.010	1.44 (0.48–4.29)	0.517
Intensity (epg)						
0	114 (93.4%)	8 (6.6%)	1.00		1.00	
1–99	104 (86.7%)	16 (13.3%)	2.19 (0.90–5.33)	0.084	1.48 (0.45–4.84)	0.520
100–399	34 (73.9%)	12 (26.1%)	5.03 (1.90–13.3)	0.001	3.21 (0.76–13.63)	0.114
≥400	27 (81.8%)	6 (18.2%)	3.17 (1.01–9.89)	0.047	0.69 (0.10–4.82)	0.706
Any helminth						
Negative	49 (94.2%)	3 (5.8%)	1.00		1.00	
Positive	230 (85.5%)	39 (14.5%)	2.77 (0.82–9.33)	0.100	0.94 (0.19–4.56)	0.935

Note: Multivariate analysis adjusted by age, gender, number of stool samples analyzed, and smoking status.
